# The Butyrogenic and Lactic Bacteria of the Gut Microbiota Determine the Outcome of Allogenic Hematopoietic Cell Transplant

**DOI:** 10.3389/fmicb.2020.01642

**Published:** 2020-07-22

**Authors:** Christian Albert Devaux, Matthieu Million, Didier Raoult

**Affiliations:** ^1^Aix-Marseille Univ, IRD, APHM, MEPHI, IHU-Méditerranée Infection, Marseille, France; ^2^Centre National de la Recherche Scientifique (CNRS), Marseille, France

**Keywords:** microbiota, butyrate, lactase, lactose, cross-feeding, graft versus host disease, hematopoietic cell transplant

## Abstract

Graft versus host disease (GVHD) is a post-transplant pathology in which donor-derived T cells present in the Peyer’s patches target the cell-surface alloantigens of the recipient, causing host tissue damages. Therefore, the GVHD has long been considered only a purely immunological process whose prevention requires an immunosuppressive treatment. However, since the early 2010s, the impact of gut microbiota on GVHD has received increased attention. Both a surprising fall in gut microbiota diversity and a shift toward Enterobacteriaceae were described in this disease. Recently, unexpected results were reported that further link GVHD with changes in bacterial composition in the gut and disruption of intestinal epithelial tight junctions leading to abnormal intestinal barrier permeability. Patients receiving allogenic hematopoietic stem cell transplant (allo-HCT) as treatment of hematologic malignancies showed a decrease of the overall diversity of the gut microbiota that affects *Clostridia* and *Blautia* spp. and a predominance of lactic acid bacteria (LAB) of the *Enterococcus* genus, in particular the lactose auxotroph *Enterococcus faecium*. The reduced microbiota diversity (likely including Actinobacteria, such as *Bifidobacterium adolescentis* that cross feed butyrogenic bacteria) deprives the butyrogenic bacteria (such as *Roseburia intestinalis* or *Eubacterium*) of their capacity to metabolize acetate to butyrate. Indeed, administration of butyrate protects against the GVHD. Here, we review the data highlighting the possible link between GVHD and lactase defect, accumulation of lactose in the gut lumen, reduction of Reg3 antimicrobial peptides, narrower enzyme equipment of bacteria that predominate post-transplant, proliferation of *En. faecium* that use lactose as metabolic fuels, induction of innate and adaptive immune response against these bacteria which maintains an inflammatory process, elevated expression of myosin light chain kinase 210 (MLCK210) and subsequent disruption of intestinal barrier, and translocation of microbial products (lactate) or transmigration of LAB within the liver. The analysis of data from the literature confirms that the gut microbiota plays a major role in the GVHD. Moreover, the most recent publications uncover that the LAB, butyrogenic bacteria and bacterial cross feeding were the missing pieces in the puzzle. This opens new bacteria-based strategies in the treatment of GVHD.

## The Gut Microbiota

**Figure 1:**
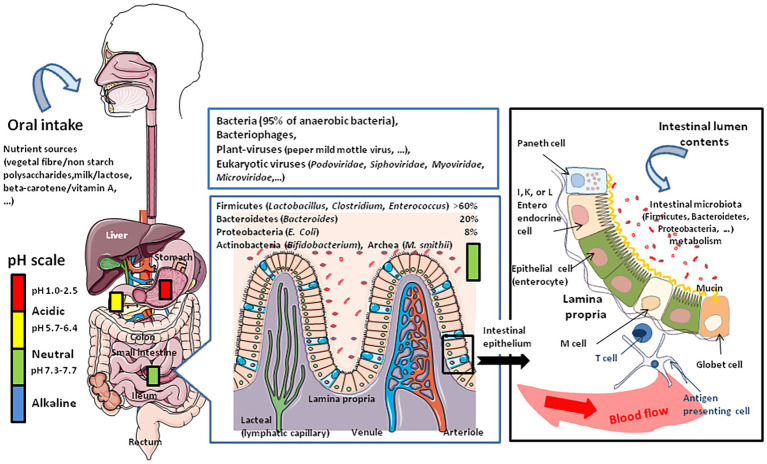
Schematic representation of oral intake including milk that contains water, vitamins, minerals, and biologically active molecules such as adipokines, leptin, adiponectin, and growth factors such as epidermal growth factor and fibroblast growth factor 21 (FGF21). The nutriments first meet the stomach microenvironment. The figure illustrates the variations in pH with marked changes from acidic in the stomach to almost neutral or slightly alkaline in the intestine. The diversity of microbiota increases from the stomach to duodenum, small intestine, and colon, where the concentration of bacteria is the highest. The abundance of microbes is about 10^1^–10^3^ colony forming unit (CFU) per gram in the stomach and duodenum, 10^4^–10^7^ CFU/g in the small intestine, and 10^10^–10^11^ CFU/g in the colon. The **middle panel** illustrates the microbiome and virome diversity in the intestinal microenvironment. The **right panel** illustrates the physiological organization of the intestinal epithelium during homeostasis. The epithelium (composed of epithelial cells) is protected by a mucus layer synthesized by goblet cells which secrete the mucins (e.g., MUC2 – mucin gel). The mucus layer allows nutrients to transport to epithelial cells but prevents bacteria attachment. The commensal intestinal microbiota is limited to the epithelium-distal mucus layer, while the epithelium-proximal mucus is largely devoid of bacteria. Intestinal bacteria such as *Bacteroides* and *Clostridia* ferment lactose into gases, hydrogen carbon dioxide, and methane when the microbiota contains an abundance of Archaea.

About 100 trillion bacteria present in the intestinal lumen (especially the colon) compose the human gut microbiota. During the last two decades, the advanced methods of high throughput sequencing and culturomics (Lagier et al., 2016) have highlighted the enormous diversity of bacteria found in humans. With its considerable bacterial genetic diversity, over 1,000 species and 7,000 strains identified, the human gut microbiota is a quite complex ecosystem, in which the phyla Firmicutes (species such as *Lactobacillus*, *Enterococcus*, and *Clostridium*) and Bacteroidetes (species such as *Bacteroides*) account for the majority of species. Other phyla including Proteobacteria (*Escherichia coli*), Actinobacteria (Bifidobacteria), Cyanobacteria, Fusobacteria, and Verrucomicrobia are also present in lower abundance (Figure 1; Eckburg et al., 2005; Qin et al., 2010). Microorganism DNA represents about 90% of the total DNA found within human bodies (Human Microbiome Project Consortium, 2012). The capacity to rapidly identify a large number of bacterial species in human microbiota opened the way to compare the gut bacterial composition in cohorts of individuals with metabolic or infectious diseases to healthy controls in search of beneficial and non-beneficial bacteria (Million et al., 2016; Tidjani Alou et al., 2017; Elkrief et al., 2019).

During the embryo development (between the first and third trimester of pregnancy), there are shifts in maternal microbiota composition, which likely provide advantages to the fetus survival (Koren et al., 2012). It is usually admitted that the microbial colonization occurs first in the amniotic fluid and placenta, and then in the maternal gut microbiota which supports the development of a prenatal microbiota in the fetus (Collado et al., 2016). When the *in utero* development is achieved, the fetus migration through the vagina favor a bacterial transfer between the microbiota colonizing the birth canal of the mother and the fetus. In favor of an early bacterial colonization of the fetus, it was observed that *Es. coli*, *Enterococcus faecalis*, and *Staphylococcus epidermidis* were isolated from the meconium of healthy neonates (Nicholson et al., 2012). The neonate survival and growth next depends on mother milk feeding that is his/her essential source of nutrients and that contributes to shape his/her gut microbiota. During the early period of breastfeeding, the infant’s gut is characterized by a low microbiota diversity and become colonized by beneficial bacteria such as *Bifidobacterium*, *Lactobacillus*, and *Prevotella* (Rautava et al., 2012; Arrieta et al., 2014). The mother milk contains about 70 g/L (7%) of lactose produced by the mammary gland (lactose synthesis requires the enzyme galactosyl transferase, which combines activated uridine di-phosphate galactose with glucose), as well as numerous biologically active factors including growth factors. The lactose is well digested by newborns whose small intestinal brush border enterocytes produce lactase in abundance. Dietary intake and bile acids (steroid acids produced in the liver and whose main function is to facilitate the absorption of fat-soluble vitamins and cholesterol) determine at least in part the microbiota assembled during the first few years of life and a shift is observed in favor of anaerobic bacteria that contribute to produce many metabolites of fermentation such as production of short-chain fatty acids (SCFAs; e.g., acetate, propionate, and butyrate; Shanahan et al., 2017). By a mechanism of cross feeding, the intestinal symbiotic microbiota contributes to maintain the production of butyrate by butyric acid bacteria; they also participate to the inhibition of pathogens growth by competing consumption of nutriments and allow to prevent toxin translocation by decomposing metabolic carbohydrates to obtain SCFAs. These SCFAs act on enteroendocrine cells of the gut through heterotrimeric guanine nucleoside-binding protein (G-protein)-coupled receptors that secrete a variety of bioactive compounds.

Lactase production decrease in the majority of the world population after weaning and most healthy adults (67–75%) produce less, sometimes very little lactase (about 10% of the concentration found of neonatal levels), whereas 25–33% retain the ability to digest lactose into adulthood (Troelsen et al., 1997). The study of ileostomy effluent samples from adult patients provided evidence that the small intestine metagenome is enriched in genes related to carbohydrate metabolism compared to the fecal metagenome (Zoetendal et al., 2012), suggesting that carbohydrate metabolism is a central function of the small intestine with lactase and propionate fermentation activities encoded by many taxa from the ileal effluent, in particular *Streptococcus* that help the growth of secondary fermenters (e.g., *Veillonella* and *Clostridium*). It is also admitted that the small intestine microbiota is phylogenetically less diverse but more dynamic than that of the colon (Booijink et al., 2010). Many bacteria living in the intestinal tract such as *Lactobacillus* sp., *Bifidobacterium* sp., *Bacillus* sp., and *Es. coli* produce lactase and play a major function in lactose absorption in the colon (Rhimi et al., 2009; Juajun et al., 2011). Dietary fibers (non starch polysaccharides) escape digestion in the human small intestine and are then broken down into simple sugars by anaerobic bacteria in the caecum and colon. The colon is more equipped to degrade complex carbohydrates and its main function is absorption of water and electrolytes and storage of fecal matter before expulsion (Turnbaugh et al., 2010). Diet degradation and absorption is under control of metabolic processes that largely depend on expression of bacterial enzymes, which are a direct reflection of the gut microbiota. The intestinal microbiota produces essential vitamins such as vitamin K, B1, B6, B9 (folic acid), and B12. Bacteria from the Firmicutes, Bacteroidetes, and Actinobacteria phyla play a role in bile acids metabolism through bile salt hydrolase activity, which catalyzes the deconjugation of conjugated bile acids (cholesterol derivatives synthesized in the liver) to liberate free primary bile acids, upregulates mucosal defenses, and controls the cholesterol homeostasis (Jones et al., 2008).

It is currently well established that homeostasis and dysbiosis are largely influenced by the composition of gut microbiota and the balance existing between different strains of bacteria (Hooper and Macpherson, 2010; Littman and Pamer, 2011). Environmental selection pressure (e.g., over‐ or under-nutrition, and antibiotics; World bank, 2017; Vieco-Saiz et al., 2019) and competitive exclusion between bacteria (e.g., probiotics and pathogenic bacteria) are expected to be the major driving forces that shape the bacterial composition of the human gut microbiota (Walter and Ley, 2011; Devaux and Raoult, 2018). Here, we provide evidence that the microbiome (the microbiota and the bacteria enzymatic equipment-driven metabolome) determine the outcome of graft versus host disease (GVHD).

## Impact of the Gut Microbiota on GVHD

If, for certain human diseases, the pathological process linked to change in the composition of the gut microbiota begins to be understood, for others it remains to be elucidated. Hematopoietic stem cell (HSC) transplantation remains indispensable for the treatment of several malignant disorders (Korbling and Freireich, 2011). Before the graft infusion, most protocols require killing of malignant cells (by chemotherapeutic drugs and/or radiotherapy myelosuppressive treatment) that cause a cytotoxic burst of tumor and normal immune cells associated with a pro-inflammatory status (Blazar et al., 2012). Then, cellular reconstitution is achieved by transplantation of peripheral blood stem cells that is the preferred source of allogenic HSC in adults in order to replace the hematopoiesis of the recipient by that of the donor. Next, it is of major importance to avoid graft rejection (mediated by the recipient immune cells) using immunosuppressive drugs. The differentiated immune cells from donor origin colonize the body (including the gut) with a risk of GVHD. The immunosuppressive therapies practiced in these patients incompletely control GVHD and increase susceptibility to infections. GVHD is considered to be mediated by activated CD8^+^ cytotoxic T-cell (CTL) from the graft donor after these cells had met graft recipient alloantigens in the context of antigen presenting cells (APCs) in the subepithelial dome of gut Peyer’s patches, the major sites where immune response is set up against luminal antigens and microorganisms (Murai et al., 2003). About 40–50% patients experience severe gastrointestinal damages from acute GVHD that turn to be fatal in about 15% of allo-transplant recipients refractory to standard steroid therapy (Blazar et al., 2012; Köhler and Zeiser, 2019). The follow-up of chimerism in patients after allo-HCT allows quantification of the donor or recipient origin of cells obtained from blood or bone marrow samples, and the chimerism is considered complete when 95% of cells are phenotypically of donor origin.

Beside the immunological activation that has long been studied in allo-transplantation, the impact of gut microbiota on GVHD has received increasing attention over the recent years. Indeed, bacterial lipopolysaccharide (LPS) released from injured gut during the condition regiment taken by the patient was considered as responsible for initiating an innate immune response through activation of toll-like receptors (TLRs) and production of cytokines, serving as a breeding ground for the onset of GVHD (Hill et al., 2000). In humans, the treatment of hematologic malignancies by allo-HCT was characterized by a marked decrease of the overall diversity of the gut microbiota, *Enterococcus* domination, and the patients with the lowest gut microbiota diversity were those with the higher mortality outcomes (Taur et al., 2012, 2014; Andermann et al., 2018). Thus, the pathophysiological mechanisms implemented in the context of a post-transplant GVHD accompanied by a modification of the gut microbiota deserve further investigation. Early administration of large spectrum antibiotics depleting *Blautia* spp. (and at a lesser extend *Clostridia*) was associated with increased GVHD and higher mortality, whereas increased abundance in *Blautia* spp. improves survival (Jenq et al., 2015). It was next confirmed that the abundance of *Clostridia* decreased in the microbiota of allo-transplant patients that experienced GHVD and was accompanied by alteration in gastrointestinal microbiota-derived butyrate (Mathewson et al., 2016). Metabolites, such as 3-indoxyl sulfate that originates from the degradation of dietary protein-derived tryptophan to indole by the tryptophanase of intestinal commensal bacteria and that is known to enhance epithelial barrier integrity and to reduce inflammation (Bansal et al., 2010), may serve as a urine marker for monitoring GVHD since microbiota perturbation in patients with GVHD is associated with lower urine levels of 3-indoxyl sulfate (Weber et al., 2015, 2017). Although bacterial populations such as *Streptococcus*, *Gemella*, and *Veillonella* considered as genera defining the core oral microbiota were little affected by the allo-HCT, at the bacterial species level, it was reported that the oral microbiota was affected in patients who developed respiratory complication after allo-HCT (e.g., decrease in *Streptococcus infantis* and increase in *Veillonella parvula*; Ames et al., 2019). Since microbiota is connected and dysbiosis is likely to be generalized. The oral microbiota could therefore be considered as a new biomarker to monitor the allo-HCT evolution.

Recently, three independent research groups decipher the molecular mechanism linking GVHD to changes in the species composition of the gut microbiota (Golob et al., 2019; Nalle et al., 2019; Stein-Thoeringer et al., 2019). The analysis of the gut microbiota of a cohort of patients with allo-HCT revealed an increase in *Enterococcus faecium*, a lactose auxotroph bacteria (which requires lactose for its *in vitro* growth), in the patient’s gut microbiota along with inflammation and intestine damages (Stein-Thoeringer et al., 2019). A decrease in *Clostridium* spp. accompanied by a significant reduction of fecal butyrate was also reported in the allo-HCT patients. This corroborate the results from Golob et al. (2019) who reported that a decrease in butyrogenic bacteria (e.g., *Blautia* spp.) in allo-HCT patients favor the GVHD, whereas administration of butyrate provide protection against GVHD.

Stein-Thoeringer et al. (2019) found that the sugar metabolism of the allo-HCT patients was impaired with an over-expression of enzymes involved in the degradation of lactose and galactose and that patients bearing a genetic polymorphism which decreases lactase expression (an enzyme also named β-d-galactosidase that is synthesized by enterocytes of the small intestine that break down the disaccharide d-lactose into d-galactose and d-glucose monomers) suffer from lactose absorption failure. It was hypothesized that lactic acid bacteria (LAB) *En. faecium* may possibly mediate the pro-inflammatory process (Zitvogel and Kroemer, 2019). Although patients with GVHD have increased intestinal permeability, the distribution of zonula occludens-1 (ZO-1) and actin were found unaltered and, in most cases, epithelial damages are limited to apoptosis of crypt epithelial cells associated with an over-expression of myosin light chain kinase 210 (MLCK210) and increased myosin II regulatory light chain phosphorylation (Nalle et al., 2019). Non-muscle myosin II (NMII), notably NMIIA, a key Rho kinase target, plays a role in epithelial cell-cell adhesion by controlling the local E-cadherin accumulation at the cell-cell contact (Priya et al., 2015). Moreover, E-cadherin is known for being used as target receptor for several bacteria and cleavage of E-cadherin by sheddases followed by the release of soluble E-cadherin is a mechanism frequently involved in disruption of the intestinal epithelium and invasive bacteria transmigration (Devaux et al., 2019). A higher risk of blood stream infection caused either by vancomycin-resistant *Enterococcus* or Gram-negative bacteria (e.g., *Es. coli* or *Klebsiella pneumoniae*) was reported, and post-engraftment vancomycin-resistant *Enterococcus* colonization was associated with increased mortality (Ford et al., 2017; Tamburini et al., 2018; Zama et al., 2020).

## The Butyrate Pathway

**Figure 2:**
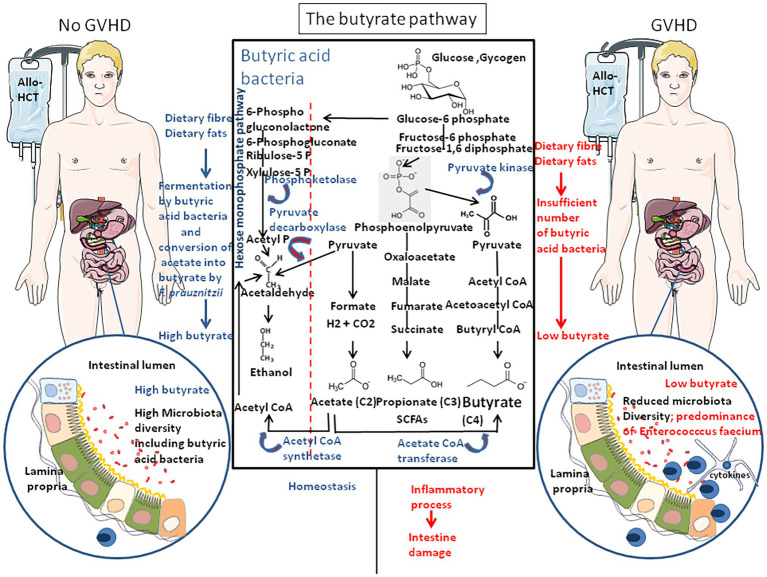
The butyrate pathway. Most of the dominant species of human colon microbiota produce acetate during the fermentation of carbohydrates. Acetate, propionate, and butyrate are the main short-chain fatty acids (SCFAs) found in humans. The main pathway for acetate formation is the oxidative decarboxylation of pyruvate leading to the synthesis of an ATP molecule. In the human colon, propionate is mainly produced by the succinate decarboxylation pathway. Propionate is mainly synthesized by the species of the genus *Bacteroides*, *Propionibacterium*, and *Veillonella*. However, the synthesis of propionate *via* lactate (not shown), could also be important, especially in the case of diets rich in non-digestible oligosides. *Clostridium*, *Eubacterium*, *Faecalibacterium*, *Roseburia*, and *Butyrivibrio* have been shown to be capable, *in vitro*, of producing butyrate by the condensation of two molecules of acetyl-CoA with synthesis of one molecule of ATP. Acetyl-CoA is also the precursor of the synthesis of ethanol, produced *in vitro* by many species such as those of the genera *Bacteroides*, *Lactobacillus*, or *Bifidobacterium*. *Faecalibacterium prauznitzii* was found able to use acetate as a source of butyrate. Butyrate is a major metabolite in colonic lumen arising from bacterial fermentation of dietary fiber and has been shown to be a critical mediator of the colonic inflammatory response. Butyrate is a source of energy and it plays a critical role in homeostasis through its ability to counteract inflammation-mediated ulcerative colitis. Patients receiving allo-HCT as treatment for hematologic malignancies and who have an abundance of butyrate-producing bacteria **(left)** have the highest probability to get reed of carbon-dioxide (GVHD), whereas those with low abundance of butyric acid bacteria **(right)** are more often faced to GVHD.

Recently, Golob et al. (2019) reported that the butyrogenic bacteria *Blautia* spp. were less abundant in allo-HCT who experienced GVHD, whereas administration of butyrate provided protection against GVHD. A decrease of fecal butyrate was also reported by Stein-Thoeringer et al. (2019) in the allo-HCT patients who experienced GVHD (Figure 2).

Butyrate, considered a protective molecule against inflammation is the end-product of anaerobic bacteria fermentation of non-digestible carbohydrates and also a component of dairy products (e.g., butter, milk, and cheese). The butyric acid bacteria are ubiquitously present in the gut microbiota of healthy humans. Oxidative stress sensitive bacteria include the butyrate producers, which mainly belong to the Firmicutes, Lachnospiraceae, Ruminococcaceae (e.g., *F. prausnitzii*, *Roseburia intestinalis*, *Eubacterium* spp., and *Coprococcus* spp.), and mucosa associated symbionts such as *Lactobacillus* spp. or *B. adolescentis*. These bacteria contribute to absorb dietary fats and lipid-soluble vitamins and facilitate lipid absorption and can digest polysaccharide dietary fiber and oxidize sugar to pyruvate. Different functionally distinct groups of butyrate-producing bacteria are present in the human large intestine: strains of *R. intestinalis* and *F. prausnitzii* possess butyryl CoA: acetate CoA transferase and acetate kinase activities but lack butyrate kinase, whereas the strain L2–50 of *Coprococcus* sp., possesses butyryl CoA: acetate CoA transferase, acetate kinase, and butyrate kinase (Duncan et al., 2002). This observation corroborates old studies performed on anaerobic butyric acid bacteria of cattle rumen that already demonstrated that among 48 strains, about 50% were able to hydrolyze starch and the number of strains that fermented certain carbohydrates was glucose, 48; esculin, 46; xylose, maltose, and cellobiose, 44; sucrose and salicin, 43; fructose, 42; lactose and inulin, 40; dextrin, 37; xylan, 34; trehalose, 16; and mannitol, 1, indicating major strain differences in genes coding for their enzyme equipment (Bryant and Small, 1956).

During the fermentation process, they produce SCFAs: acetate (C2), propionate (C3), and butyrate (C4). The SCFAs concentration is 10 times higher in the colon than in the ileum (Cummings et al., 1987). Acetate, propionate, and butyrate represent between 85 and 95% of total SCFAs found in the host and the molar ratio acetate/propionate/butyrate is about 60/20/20 (Cummings and Macfarlane, 1991; Topping and Clifton, 2001). About 95% of the SCFAs produced in the colon are absorbed from the colonic epithelium and 5–10% of the SCFAs are excreted by the feces (Hijova and Chmelarova, 2007). Acetate and propionate are transported through the blood to a variety of organs, where they are substrates for oxidation, lipid synthesis, and energy metabolism. Acetate is mainly metabolized by liver cells (50–70%), but also by the heart and skeletal muscles and the brain, making it an important source of energy. Propionate is also metabolized by the liver and used by hepatocytes for glucogenesis (Cummings et al., 1987; Nicholson et al., 2012). Butyrate is used by the cells of the colon and liver and targeted to their mitochondrial compartment for energy purposes (Clausen and Mortensen, 1994; Basson et al., 2000; Della Ragione et al., 2001).

Most of the dominant species of human colon microbiota produce acetate during the fermentation of carbohydrates. The main pathway for acetate formation is the oxidative decarboxylation of pyruvate leading to the synthesis of an ATP molecule. In the human colon, propionate is mainly produced by the succinate decarboxylation pathway (Miller and Wolin, 1979). Propionate is mainly synthesized by the species of the genus *Bacteroides*, *Propionibacterium*, and *Veillonella*. However, the synthesis of propionate *via* lactate could also be important, especially in the case of diets rich in non-digestible oligosides. Butyrate is mainly produced by distinct families within the Firmicutes, Ruminococcaceae, and Lachnospiraceae. But members of other phyla including Bacteroidetes, Actinobacteria, Fusobacteria, and Proteobacteria are potential butyrate producers (Vital et al., 2014; Zhang et al., 2019). Bacteria that belong to *Clostridium*, *Eubacterium*, *Faecalibacterium*, *Roseburia*, and *Butyrivibrio* have been shown to be capable, *in vitro*, of producing butyrate by the condensation of two molecules of acetyl-CoA with synthesis of one molecule of ATP. Most of the bacterial diversity in bacteria that produce butyrate by the acetyl-CoA pathway is associated with Ruminococcaceae and Lachnospiraceae, whereas other bacteria such as Bacteroidetes mainly use other (Glutarate, Lysine) pathways (Vital et al., 2014). Acetyl-CoA is also the precursor of the synthesis of ethanol, produced *in vitro* by many species such as those of the genera *Bacteroides*, *Lactobacillus*, or *Bifidobacterium*. Pyruvate is a central compound of the butyrate pathway and in turn can be oxidized to acetyl CoA with production of hydrogen, methane, and carbon dioxide gases (CO_2_ increases the pH) and energy, which the bacteria require for growth and production of many molecules. Part of acetyl CoA is converted into acetic acid with ATP production and butyryl CoA that is metabolized into butyrate with ATP production. In acetate-rich diet, where acetate was delivered by resistant starch directly into the gastrointestinal tract, butyric acid bacteria can convert both exogenous and gut microbiota-produced acetate into butyrate (a reaction that requires acetate CoA transferase and butyrate kinase). One of the rare known bacteria that can achieve this enzymatic reaction is *F. prausnitzii* (Duncan et al., 2002), thereby contributing to the protection of the intestinal mucosa barrier and to the promotion of *Bifidobacterium* and *Lactobacillus* improving intestinal function (Si et al., 2018). *F. prausnitzii* grows poorly in culturomic medium that does not contain acetate and this requirement likely explains its dependence on rumen fluid. SCFAs are able to bind and activate the G-protein coupled cell surface receptors (including FFA2/GPR43 and FFA3/GPR41) expressed by enteroendocrine I, K, and/or L epithelial cells that are known to play an important role in the regulation of glucose homeostasis and appetite (Jorsal et al., 2018; Priyadarshini et al., 2019). In a murine model, it was shown that FFA2/GPR43 is also strongly expressed in a large population of leukocytes in the lamina propria; that FFA3/GPR41 is expressed in subpopulations of ghrelin and gastrin cells in the stomach, in secretin cells of the proximal small intestine, in GLP-1, YY, and neurotensin cells of the distal small intestine and proximal colon; and that a gradient of FFA3/GPR41 expression exists among the somatostatin cells from less than 5% in the stomach to more than 95% in the rectum (Nohr et al., 2013). FFA2/GPR43 has similar affinity for acetate, propionate, and butyrate whereas FFA3/GPR41 has preferential affinity for propionate, although it also binds butyrate. In a murine model of GVHD, mice orally force-fed with GPR43 antagonist GLPG0974 (10 mg/kg/day) demonstrated significant higher GVHD than control, indicating a protective effect against GVHD when butyrate can bind FFA2/GPR43 (Fujiwara et al., 2018). Beside being a major source of energy for the intestinal epithelium (this allow energy-deprived cells to escape autophagy) and source of nutriment for microbes such as *Desulfotomaculum* spp. (Kuwahara, 2014), butyrate acts on the epigenetic regulation of genes by inhibiting an histone deacetylase (HDAC; Waldecker et al., 2008) and as an agonist for peroxisome proliferator-activated receptors (PPARs) that control both lipid metabolism and inflammation (Varga et al., 2011). Butyrate increases expression of proteins (such as junctional adhesion molecules/JAM/occludin) involved in the stability of tight junctions in colon epithelia; it regulates the neutrophil function and migration and inhibits inflammatory cytokine-induced expression of vascular cell adhesion molecule-1 (V-CAM1). It can pass through the enterocytes into the circulation. Butyrate and retinoic acid (RA) co-operate to regulate the innate immune response. Vitamin A is taken from food in the form of retinol, retinoic acid, or beta-carotene. In the gut, dendritic cells (DCs) metabolize vitamin A in RA, and RA co-operates with butyrate to induce mucosal-like CD103^+^DCs differentiation required to trigger the differentiation and intestinal recruitment of FoxP3^+^ T regulator (T reg) cells, IgA antibody secretion, and reduce inflammation (Qiang et al., 2017). The butyrate receptors, FFA2/GPR43 and FFA3/GPR41, are all found expressed on liver cells. At high concentration, butyrate is expected to promote anti-tumor effect by optimizing the effector function of CD8^+^ T cells that produce IFN-γ (Luu et al., 2018).

## Radiotherapy, Antibiotics/Chemotherapy, and Oxidative Stress-Induced Loss of Cross Feeding Bacteria

Radiotherapy and antibiotic/chemotherapy treatments that are practiced to kill fast dividing cancer cells and as treatment of complications of allo-HCT are known to induce oxidative stress, where high levels of reactive oxygen species (ROS) and reactive nitrogen species (NOS) are generated. The free oxygen radicals hydroxyl radical (OH), superoxide anion (O_2_−), hydrogen peroxide (H_2_O_2_) trigger the up-regulation of cyclooxygenases (COX), nitric oxide synthase, lipoxygenases, and nicotinamide adenine dinucleotide phosphate oxidase, leading to DNA damage, inflammation (radiation-induced enteritis/radiation-induced bowel injury), cell apoptosis, and also modify the microbiota homeostasis (Saha et al., 2017; Deleemans et al., 2019; Severyn et al., 2019). In a non-human primate model, ionizing radiations were also reported to induce up-regulation of tumor necrosis factor alpha (TNFα) and metalloprotease MMP7 (Zheng et al., 2015), likely affecting the intestinal epithelium barrier integrity and bacterial infiltration.

**Figure 3:**
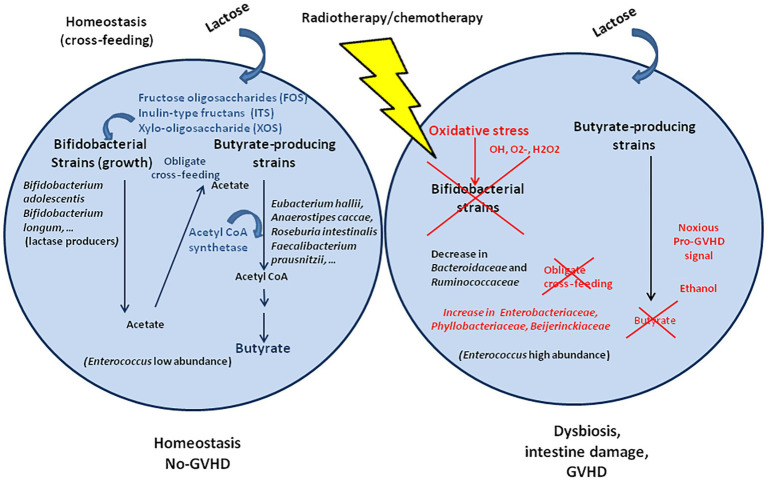
Cross-feeding that take place between *Bifidobacterium* spp. and species of butyrate-producing colon bacteria. Within the microbiota, the bacteria equipped with superoxide dismutase and catalase anti-oxidant enzymes can be resistant to the free radical produced after radiotherapy/chemotherapy, whereas bacteria that lack these enzymes are highly sensitive to oxidative stress. *Bifidobacterium* that are essential to fermentation are not able to produce butyrate but produce acetate to cross-feed the butyrate producing colon bacteria. Under oxidative stress, the *Bifidobacterium* are killed and no longer play their role as cross-feeding bacteria with direct consequences on the production of butyrate by the butyrogenic bacteria.

Radiation enteritis and dysbiosis were reported in patients who received a radiotherapy treatment. The dysbiosis was characterized by a relative higher abundance of Proteobacteria and Gammaproteobacteria and lower abundance of *Bacteroides*. A deeper analysis found an increase in oxidative stress resistant Enterobacteriaceae, Phyllobacteriaceae, and Beijerinckiaceae, whereas oxidative stress sensitive Bacteroidaceae and Ruminococcaceae were decreased (Figure 3; Wang et al., 2019). It confirms previous observation from radiation-induced intestinal chronic inflammation (Kumagai et al., 2018). Within the microbiota, some bacteria are resistant to oxidative stress (bacteria that synthesize the anti-oxidant enzyme superoxide dismutase, SOD, which neutralize O_2−_, and catalase, and CAT, which neutralize H_2_O_2_), whereas others are highly sensitive. *Bifidobacterium* that are essential to fermentation are not able to produce butyrate although they are associated to a butyrogenic effect due to cross-feeding between *Bifidobacterium* and butyrate producing colon bacteria (Falony et al., 2009; De Vuyst and Leroy, 2011). *Bifidobacterium* are preferentially stimulated to growth in the presence of fructose oligosaccharides (FOS), inulin-type fructans (ITF), and xylo-oligosaccahride (XOS; Tuohy et al., 2001; Roberfroid, 2007; Lecerf et al., 2012). Cross-feeding between starch-degrading Bifidobacteria and lactate-converting, butyrate‐ producing colon bacteria (e.g., *Eubacterium hallii* and *Anaerostipes caccae*) has been demonstrated (Duncan et al., 2004). Indeed, *Eu. hallii* L2-7 and *A. caccae* L1-92 failed to grow on starch in pure culture, but in co-culture with *B. adolescentis* L2-32, butyrate was formed, indicating cross-feeding of metabolites to the lactate user bacteria (Belenguer et al., 2006). Obligate cross-feeding was also reported between *B. longum* BB53 and *A. caccae* DSM 14662 and *R. intestinalis* DSM 14610, *B. longum* acting as acetate producer for the butyrogenic bacteria (Falony et al., 2006), and in co-cultures of *B. adolescentis* L2-32 with *F. prausnitzii* S3/L3 or *F. prausnitzii* A2-165 with FOS as carbon source, resulting in acetate decrease and butyrate increase (Rios-Covian et al., 2015). Metabolic cross-feeding can occur *via* intercellular nanotubes among bacteria (Pande et al., 2015).

In the presence of carbohydrates, *Bifidobacterium* cross feed the butyrogenic bacteria that in turn produce butyrate (Rivière et al., 2016). However, *Bifidobacterium* strains such as *B. adolescentis* are highly sensitive to oxidative stress. It is therefore possible that during the chemotherapy that is practiced in patients with allo-HCT, such bacteria are lost. In methotrexate chemotherapy of lymphoblastic leukemia, a significant reduction of *Bifidobacterium*, *Lactobacillus*, and *Es. coli* was reported (Huang et al., 2012). Without cross-feeding, the production of butyrate will be drastically reduced. Treatment of patients with melatonin (*N*-acetyl-5-methoxytryptamine know to exhibit antioxidant activity) post-irradiation significantly increased both SOD and CAT, suggesting that the treatment restore the oxidative stress resistant bacteria (Musa et al., 2019).

Taken together, these results suggest that digestive bacteria sensitive to oxidative stress, capable of catabolizing lactose and increasing butyrate production are lost in GVHD. It was reported that the use of *Lactobacillus rhamnosus* (a butyric bacteria species) could prevent the occurrence of diarrhea in patients receiving radiotherapy (Delia et al., 2002). Other studies reported that use of *Lactobacillus acidophilus* and *Bifidobacterium bifidum* were beneficial to the patients, yet, the results remain controversial (Kumagai et al., 2018; Wei et al., 2018), likely because these bacteria display phenotypic variation on strain level and each clone is different in terms of enzymatic equipment. More recently, it was reported that fecal microbiota transplant (FMT) improves the outcomes of an allo-HCT with GVHD (Severyn et al., 2019).

## The Lactase Pathway

**Figure 4:**
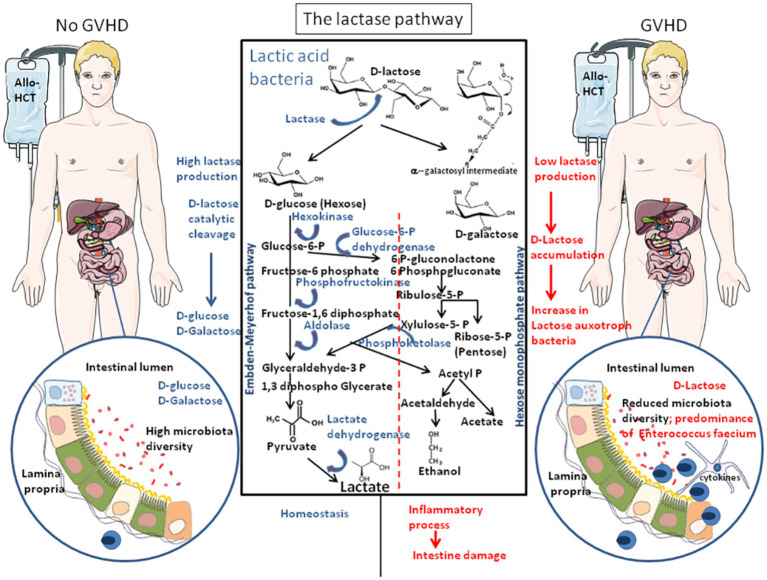
The lactase pathway. The FGF21 was found to enhance MAPkinases (ERK1/2) phosphorylation in intestinal explants and to stimulate lactase production and glucose homeostasis. The lactic acid bacteria (LAB) of the intestinal microbiota are the main source of lactase in adult. Bacterial lactase genes in the intestinal microbiota derived from Actinobacteria, Proteobacteria, and Firmicutes. The bacterial species that transform the carbohydrates into lactate mainly belong to the genera *Bifidobacterium* and *Lactobacillus*, as well as *Streptococcus* and *Enterococcus*. Lactate is produced by reduction of pyruvate. LAB can be divided in two metabolic classes: first, the homofermentative bacteria that produce lactate includes some Lactobacilli and most species of *Pediococcus*, Vagococci, Streptococci, Tetragenococci, and Enterococci (including *Enterococcus faecium*), and the heterofermentative species include Leuconostocs, Oenococci, *Weissella* species, and some lactobacilli that produce lactate, carbon-dioxide (CO_2_), acetate, and/or ethanol. Patients receiving allo-HCT as treatment of hematologic malignancies and who have an abundance of lactase-producing bacteria **(left)** produce lactate from lactose and are less sensitive to GVHD. Patients with allo-HCT, who carry a microbiota that produces low lactase, accumulate lactose that increases the abundance of lactose auxotroph bacteria (*En. faecium*) with a concomitant decrease of the microbiota diversity **(right)**, triggering pro-inflammatory responses, and serving as a breeding ground for GVHD.

Recently, Stein-Thoeringer et al. (2019) reported that patients with allo-HCT who experienced GVHD were characterized by an increase in the lactose auxotroph (which require lactose for their *in vitro* growth) bacteria *En. faecium* in their gut microbiota that was accompanied by inflammation and intestine damages. It was hypothesized that LAB *En. faecium* may possibly mediate the pro-inflammatory process (Figure 4; Zitvogel and Kroemer, 2019).

The intestinal microbiota is the main source of lactase in adult. Bacterial lactase genes in the intestinal microbiota mainly derived from Actinobacteria, Proteobacteria, and Firmicutes (Long et al., 2018). It is excluded to draw general conclusions on a metabolism picture that would be common for all LAB. Each species and possibly each clone may behave differently depending on their/its enzymatic equipment. LAB are Gram-positive bacteria, acid-tolerant, low G+C content in the DNA, that belong to *Lactobacillus*, *Enterococcus*, *Streptococcus*, *Pediococcus*, *Lactococcus*, and *Oenococcus* genus have the capability to modify the environment in which they are delivered (Makarova et al., 2006). LAB genomes code between 1,700 and 2,500 proteins. The growth optimum for LAB is at pH 5.5–5.8, and these bacteria have complex nutritional requirements for carbohydrates and other compounds such as fatty acids, peptides, vitamins, amino acids, peptides, nucleotide bases, and minerals. LAB produce lactase, an enzyme that catalyze the cleavage of carbohydrates (lactose, glucose, sucrose, or galactose) into lactic acid (Rhimi et al., 2009; Russo et al., 2017). Lactase is an important enzyme associated with lactose absorption. Dysbacteriosis associated to inhibition of intestinal lactase activity cause body diarrhea by affecting body’s absorption of nutriments. A variety of diarrhea can be treated by supplementing oral intake with LAB or lactase (Luo et al., 2016).

Based on sugar fermentation patterns, LAB can be divided in two metabolic classes: first, the homofermentative bacteria that include some Lactobacilli (such as *Lactobacillus casei*, *Lactococcus lactis*, and *Lactobacillus plantarum*), most species of *Pediococcus* (such as *Pediococcus pentasaceus* and *Pediococcus acidilactici*), Enterococci (such as *En. faecium*), Streptococci, Tetragenococci, and Vagococci that ferment hexoses by the Embden-Meyerhof pathway. It was reported that *Streptococcus faecalis*, that produce glucose-6-phosphate dehydrogenase and 6-phosphogluconate dehydrogenase, regulate the metabolism of glucose by the specific inhibitory interaction of the Embden-Meyerhof intermediate fructose-1,6-diphosphate with 6-phosphogluconate dehydrogenase (Brown and Wittenberger, 1971). In the presence of homofermentative LAB, lactate is the primary product with a production of two moles of lactate from one mole of glucose (homofermentative LAB are bacteria common to the dairy industry); second, the heterofermentative species include Leuconostocs, Oenococci, *Weissella* species, and some Lactobacilli (such as *Lactobacillus buchneri*). The heterofermentative LAB species produce one mole of lactate from one mole of glucose, as well as CO_2_ and acetate and/or ethanol. High amount of ethanol was shown to be produced by *Lactobacillus fermentum* by glucose metabolism and *Weissella confusa* by fructose metabolism, whereas addition of pyruvate reduced their production of ethanol with a shift to acetate production (Elshaghabee et al., 2016). LAB can also synthesize different compounds such as bacteriocins (ribosomally synthesized anti-microbial peptides), H_2_O_2_, and enzymes capable to facilitate nutriment acquisition (such as protease, amylase,…), and other enzymes (such as β-galactosidase, galactose mutarotase, l-lactate dehydrogenase,…; Liao and Nyachoti, 2017). The apparent difference between the homofermentative and heterofermentative classes of LAB is the presence or absence of the fructose-1,6-diphosphate and/or phosphoketolases. Bacteria that produce xylose isomerase and xylulokinase can convert xylose, a pentose, to xylulose 5-phosphate. In many bacteria, such as *Es. coli*, xylulose 5-phosphate is further catabolized to form glyceraldehyde-3 phosphate by the transketolase and transaldolase enzyme of the pentose phosphate pathway. Another xylose catabolic pathway used by heterofermentative bacteria such as *Clostridium acetobutylicum*, involves phosphoketolases that cleave xylulose 5-phosphate into acetyl-phosphate and glyceraldehyde-3-phosphate (Liu et al., 2012). In Bifidobacteria, phosphoketolases are key enzymes to convert fructose-6-phosphate to acetyl-phosphate and erythrose-4-phosphate (Grill et al., 1995). Recently, a *Collinsella aerofaciens* subspecies that uses butyric acid kinase and phosphatase butyryltransferase enzyme to metabolize sugars was described (Qin et al., 2019).

When produced at high concentration, lactate may in turn inhibit proline oxidase, thereby regulating the bioavailability of proline (a markedly reduced rate of proline degradation) in the liver (Kowaloff et al., 1977). A pathologic hyperprolinemia could be related to the hyperlactacidemia found in acquired lactic acidosis and alcoholic cirrhosis, and there are evidence in the literature that in infants with plasma concentration of lactate 8–10-fold normal, their plasma proline was two to three-fold the normal concentration and that patients with lactic acidosis showed a plasma concentration of proline five to six-fold the normal level. Since, proline can be a source for gluconeogenesis (a metabolic pathway that results in the generation of glucose from glucogenic amino acids, triglycerides, glycerol, pyruvate, and lactate), increased lactate concentration in the liver may trigger acute hepatic gluconeogenesis that acts as metabolic fuels.

Several reports indicate that LAB induce adaptive immune responses (Kiczorowska et al., 2017; Han et al., 2018). Feeding piglets with *L. rhamnosus* prevented acute infectious diarrhea by triggering the lamina propria CD3^+^/CD4^+^ T cells activation (Zhu et al., 2014). *L. plantarum* was shown to stimulate anti-*Salmonella* immune response in pigs (Tran et al., 2016). In chickens, feed supplementation with *L. acidophilus* increases the production of CD3^+^/CD4^+^ and CD3^+^/CD8^+^ T cells in their gastrointestinal tract and peripheral blood (Asgari et al., 2016). LAB (mainly *Lactobacillus*) are currently considered as a possible probiotics and they are intensively studied to select the most valuable strains for commercial use (Aristimuño Ficoseco et al., 2018). Thus, Lactobacilli are mainly grouped in the growth promoter beneficial bacteria.

## Possible Relationship Between Pathogenic Lactic Acid *Enterococcus* Genus and GVHD

**Figure 5:**
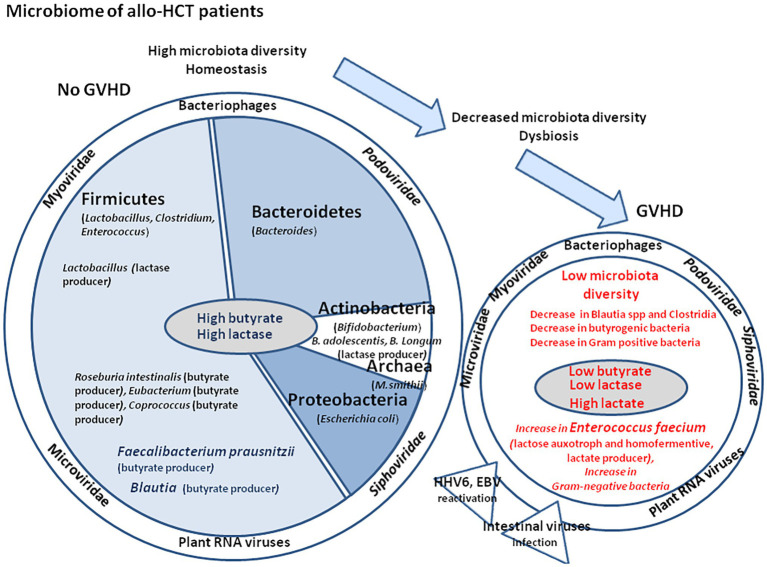
Microbiome and virome in patients with allo-HCT. Many bacteria living in the intestinal tract **(left panel)** such as *Lactobacillus* sp., *Bifidobacterium* sp., *Bacillus* sp., and *Escherichia coli* produce lactase and play a major function in lactose absorption in the colon. Bacteria from the Firmicutes, Bacteroidetes, and Actinobacteria phyla play a role in bile acids metabolism and up-regulate mucosal defenses. *Faecalibacterium prausnitzii* is one of the rare bacteria that can convert acetate into butyrate, thereby contributing to the protection of the intestinal mucosa barrier and to the promotion of *Bifidobacterium* and *Lactobacillus* improving intestinal function. The presence of *F. prausnitzii* and *Blautia* spp. in the patient microbiota is a favorable prognosis mark for the patient. During GVHD **(right panel)**, an increase in the lactose auxotroph bacteria *En. faecium* is observed along with inflammation and intestine damages. A decrease in *Clostridium* spp. and *Blautia* spp. (butyrogenic bacteria) is accompanied by a significant reduction of fecal butyrate in GVHD. Gram-positive Lactobacilli, *Clostridia*, Bifidobacteria, and *Bacillus* spp. were less abundant in GVHD. In contrast, the proportion of Gram-negative bacteria (Enterobacteriaceae, Enterococci, and *Bacteroides*/*Prevotella* spp.) increased, as well as release of lipopolysaccharide (LPS) that trigger toll-like receptor (TLR) induction and low-grade chronic inflammation. During GVHD reactivation of viruses [e.g., human herpes virus 6 (HHV-6) or EBV] is frequently observed.

Although Enterococci are part of the normal intestinal microbiota, several species have a notable clinical implication, in particular *En. faecalis* and *En. faecium*. These Enterococci species cause a variety of diseases, including endocarditis, urinary tract infections, prostatitis, and cellulitis (Noris et al., 2010; Paganelli et al., 2016; Tien et al., 2017; Beganovic et al., 2018; Reissier et al., 2018). About 3% of the *En. faecalis* and *En. faecium* genomes encode enzymes involved in lactose and galactose metabolism (Figure 5; Stein-Thoeringer et al., 2019). *En. faecium*, is known for its ability to co-metabolize citrate and lactose and to produce high amounts of lactate (by conversion of pyruvate into lactate; Cabral et al., 2007). Risk factors for colonization and infection must be recognized, in particular prior treatment with antibiotics such as cephalosporins or quinolones. The veterinary use of vancomycin and avoparcin as growth factor in the feed supplementation of farm animals is likely one of the reasons for the selection of VanA strains that resist both vancomycin and avoparcin (a resistance that is uncommon except in Enterococci; Bortolaia et al., 2015). *En. faecalis* was reported as capable of producing extracellular superoxide and H_2_O_2_ that damage colonic epithelial cell DNA (Huycke et al., 2002). Unlike most commensal strains, the genome of multidrug-resistant (MDR) strains of *En. faecalis* clinical isolates are rich in mobile genetic elements and lack genome defense system composed by the clustered regularly interspaced short palindromic repeat (CRISP) and the CRISP-associated protein (CAS; Hullahalli et al., 2018). Moreover, *En. faecalis* incorporates to its membrane host-derived fatty acids found in human serum that protect the bacteria against membrane-damaging antibiotics (e.g., daptomycin; Saito et al., 2018). Another species of Enterococci, *Enterococcus gallinarum* was found to expand in mice treated with proton pump inhibitors (PPIs; Llorente et al., 2017). This strain is known to trigger pro-inflammatory pathways, to alter gut-barrier related molecules in the small intestine, and to translocate to the mesenteric lymph node, spleen and liver, inducing systemic autoimmunity both in mice and humans (Manfredo Vieira et al., 2018). *En. gallinarum* was also found to cause endocarditis (Angelos et al., 2018).

## Microbiota and the Innate Immunity: Relationship to GVHD

Interactions between the gut microbiota and the host immune system begin at birth and these two systems are involved in a complex interplay. The crosstalk that takes place determines the host immune inflammation status. Several studies reported that innate immune cells (e.g., neutrophils and inflammatory monocytes) are recruited to the gut shortly after allo-HCT (Schwab et al., 2014).

**Figure 6:**
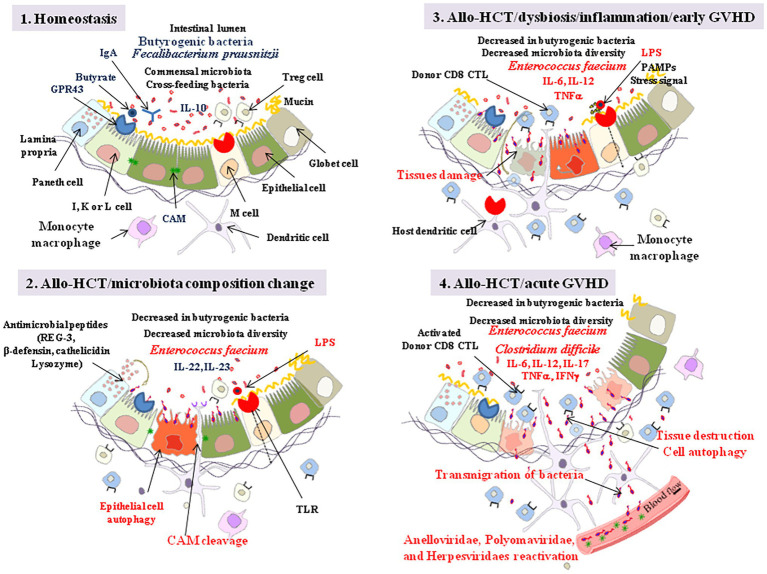
Allo-HCT: from homeostasis to acute GVHD. Step 1: intestinal lumen microenvironment characteristic of homeostasis with high butyrate, presence of FoxP3 T reg cells, and anti-inflammatory interleukin 10 (IL-10). Butyrate controls the homeostasis through interaction with GPR43 expressed by enteroendocrine I, K, and/or L cells. The engagement of GPR43 triggers ERK signaling and subsequent NOD-LRR‐ and pyrin domain-containing protein 3 (NLRP3) inflammasome activation which promotes protection of intestinal epithelial cells and repairs by increasing IL-18 secretion. Butyrate also acts as a histone deacetylase inhibitor, thereby increasing expression of anti-apoptotic genes (e.g., BCL-B, a member of the BCL-2 family). The epithelial cells maintain a barrier through intercellular junctions that involve CAM, such as E-cadherin. Step 2: following allo-HCT, when the gut microbiota suffers quantitative and qualitative changes in bacteria composition (e.g., predominance of *En. faecium*), the release of bacterial LPS is considered as responsible for initiating an innate immune response through activation of TLRs at the surface of villous microfold cells (M cells). These cells deliver the luminal antigens to the underlying immune system to set up a whole arsenal of anti-bacterial innate immunity. Indole transitorily stimulates the production of IL-22 which protects the intestinal epithelial cells. Host DCs present recipient allo-antigens to donor CD4^+^ and CD8^+^ T cells that produce pro-inflammatory cytokines, serving as a breeding ground for GVHD. Through their MR1 molecules, dendritic cells (DCs) also activate host mucosal associated invariant T (MAIT) cells to secrete IL-17A, which enhances the intestinal barrier integrity. The Paneth cells produce antimicrobial proteins (e.g., C-type lectin REG3γ, β-defensins, cathelicidins, and lysozyme). Sheddases produced by bacteria or by eukaryotic cells through bacterial signaling initiate the cleavage of cell adhesion molecule (CAM; e.g., cleavage of E-cadherin), creating damages in the intestinal barrier. Step 3: the sequential detection of stress signals, tissue damages, and pathogen-associated molecular patterns (PAMPs; e.g., lipopolysaccharide, flagellin, peptidoglycan, lipoproteins, and unique bacterial nucleic acid structures) by the host antigen presenting cells activates the immune system. KLRG1^+^ dendritic cells and monocytes/macrophages, CD103^+^ T-cells, KLRG1^+^ T-cells, and other immune cell subpopulations, colonize the lamina propria. To challenge the dysbiosis, epithelial cell autophagic clearance and dead cells renewal accelerates but remains unsuccessful due to aggravation of the pro-inflammatory process that combine activation of anti-recipient host alloantigens donor CD8^+^ cytotoxic T cells (CTL) and proinflammatory cytokines (IL-6, IL-12, TNFα) production. Alloreactive T cells from the donor attack healthy tissues in the recipient allo-HCT patient. Step 4: the inflammatory process develop accompanied with transmigration of bacteria outside the intestinal lumen (*Clostridium difficile* has been frequently found associated with severe GVHD), reactivation of viruses, further tissue destruction and global aggravation (spread to other tissues) of the disease with characteristic symptoms including abdominal cramping and diarrhea, and biological markers of abnormal liver function (elevation of alkaline phosphatase and bilirubin). GVHD can be also monitored by skin, liver, and gut biopsis. GVHD is also associated with lower urine levels of 3-indoxyl sulfate.

The gut microbiota shapes the gut mucosal immune system and the intestinal immunoglobulin A (IgA) produced by B cells predominantly target the commensal bacteria that reside in the small intestine, yet more IgA-producing B cells are recruited during inflammatory processes (Million et al., 2018). Gut homeostasis does not just rely on IgA production following microbial immune priming. Vitamin A derived RA together with butyrate were shown to maintain gut immune homeostasis through the induction of CD103^+^ DC cells (in the mesenteric lymph nodes and colonic lamina propria) and recruitment of FoxP3^+^ T reg cells (Qiang et al., 2017). The butyric acid bacteria *F. prausnitzii* (one of the most abundant Firmicutes in the gut) that are enzymatically equipped for the conversion of acetate into butyrate (it express acetate CoA transferase and butyrate kinase; Duncan et al., 2002), were found to exhibit anti-inflammatory properties through the stimulation of anti-inflammatory cytokine IL-10 expression and reduction of the pro-inflammatory cytokine IL-8 expression (Tremorali and Backhed, 2012; Heinken et al., 2014). Butyrate was also found to suppress expression of proinflammatory cytokines interleukin 6 (IL-6) and tumor necrosis factor (TNF)-α to reduce IL-12 expression, and butyrate-treated DCs showed a decreased capability in priming CD4^+^ T cell proliferation. However, SFCAs at high concentration can also induce Th1 and Th17 cells upon immunological challenge and, therefore, also have the potential to induce inflammatory responses (production of IL-17 and IFNγ; Park et al., 2020). Moreover, the induction of the pro-inflammatory cytokine IL-23 in DCs by butyrate is linked to its role as a HDAC inhibitor for epigenetic modification of genes. Through binding to the retinoic acid-receptor -γt, IL-23 induces the production of the epithelial cell regenerative factor IL-22. Altogether, these observations indicate that CD103^+^ DC cells and FoxP3^+^ T reg cells, therefore, regulate the balance between gut tolerance and intestinal inflammation (Figure 6).

A high fat diet is known to alter the composition of the intestinal microbiota, notably decreasing the number of the Gram-positive Bifidobacteria bacteria and increasing the proportion of Gram-negative bacteria in the gut, and, hence, increase the release and plasma concentration of LPS and trigger TLR induction that generates low-grade chronic inflammation (Wagnerberger et al., 2012). Indeed, the TLR^+^ villous microfold cells (M cells) present in the intestinal epithelium deliver luminal antigens to the underlying immune system either by transcytosis or microvesicle uptake, while alternatively M cell apoptosis and generation of transcellular pores through which DCs can gain access to the intestinal lumen may also allow capture of antigens and antigen presentation (Million et al., 2018). In mice model with deleted LPS receptors and CD14 mutants (CD14 is the co-receptor for the LPS-receptor TLR4), a hypersensitivity to insulin was reported, suggesting that high-fat diet-induced metabolic dysfunction occurs through the LPS/CD14 signaling pathway (Cani et al., 2007). LPS is a marker of Gram-negative bacteria death. High-fat diets have been shown to induce the passage of LPS from the intestine into the mesenteric lymphatic system (possibly through binding of chylomicrons, formed from dietary triglycerides, to LPS), leading to inflammation (Vreugdenhil et al., 2003). Production of chylomicrons was also reported capable to promote production the (TNF)-α proinflammatory cytokine. In mice models, cross linking of TLR9 by bacterial DNA stimulate GVHD whereas mutations in the *TLR9* and *TLR4* genes preventing bacterial LPS recognition were found to reduce GVHD (Calcaterra et al., 2008; Imado et al., 2010). Polymorphism of nucleotide-binding oligomerization domain 2 (*NOD2*) gene that encodes a receptor for the pathogen-associated molecular patterns (PAMPs) bacterial associated molecules is linked with a higher incidence of GVHD in HSC transplant recipients (Penack et al., 2010). The TLR5 agonist fagellin extracted from bacterial flagella was shown to reduce GVHD and preserve long post-transplant immune reconstitution characterized by more Fop-3^+^ T regulator (T reg) cells (Hossain et al., 2011). Studying the role for TLR9 and its downstream signaling adaptor MYD88 in an intestine GVHD model, Heimessat et al. (2010) observed a shift towards Enterobacteriaceae, Enterococci, and *Bacteroides*/*Prevotella* spp. being elevated in the colon lumen during GVHD, whereas Lactobacilli, *Clostridia*, Bifidobacteria and *Bacillus* spp. were less abundant. *In fine*, GVHD is mediated by CD8^+^ CTLs that express anti-host specificities. In a murine model, it was found that GVHD may be inhibited by preventing CD8^+^ CTL migration into the Peyer’s patch either by disrupting the gene encoding the CCR5 chemokine receptor or by blocking the integrin α4β7-mucosal vascular addressin (MAdCAM-1) interaction into the gut Peyer’s patches (Murai et al., 2003). Since mutants in the integrin α4β7 can also affect the integrin binding to E-cadherin (Higgins et al., 2000), it remains possible that such mutants might also affect the anti-bacterial CTL immune response through aberrant homing signals that involve E-cadherin, soluble E-cadherin, CD103, and KLRG1, as previously suggested (Devaux et al., 2019).

## Is there Room for Viruses in GVHD?

Immense populations of viruses, among which bacteriophages, are present in the human gut, and lysogenic or temperate phages are able to integrate their chromosome into the bacterial genome, sometimes altering the phenotype of host bacteria. A number of different eukaryotic viruses have also been found in the human gut virome with a predominance of the pepper mild mottle virus, a plant-infecting RNA virus derived from diet (Zhang et al., 2006). A very elegant study by Minot et al. (2011) reported the presence of *Myoviridae*, *Siphoviridae*, *Podoviridae*, *Tectiviridae*, *Inoviridae*, and *Microviridae* in human stools from healthy donors. Among double stranded DNA viruses found in the human gut, a predominance of *Podoviridae* followed by *Siphoviridae* and *Myoviridae* was reported in healthy humans while *Microviridae* dominated the single strand DNA viruses (Kim et al., 2011). Kim et al. (2011) also reported the rare presence of picornaviruses, herpesviruses, and poxviruses. Many viruses (e.g., rotaviruses, caliciviruses, astroviruses, enteric adenoviruses, toroviruses, and parechoviruses) are known to induce gastroenteritis in humans, the leading cause of diarrheal disease being rotaviruses, a *Reoviridae* (Clark and McKendrick, 2004). Therefore, it cannot be ignored that the virome could influence GVHD just as easily as the microbiome. Although eukaryotic cell-borne viruses are minor components of the human gut virome compared to the bacteriophages, latent viral infections can be reactivated in patients with allo-HCT who experience a phase of immunosuppression. Norovirus induced gastroenteritis was found to be a major threat to patients to allo-HCT (Schwartz et al., 2011). Reactivation of human herpes virus 6 (HHV-6) was reported to be a predictive factor for acute GVHD (Pichereau et al., 2012). The HHV-6 reactivation reported in 35% of patients who received an allo-HCT (sometimes associated with a subsequent cytomegalovirus reactivation) was found predictive of mortality after allo-HCT (Zerr et al., 2012). Feghoul et al. (2015) reported that human adenovirus infections constitute a major cause of morbidity in pediatric allo-HCT patients. The incidence rate at day 100 was 35.9% for the adenovirus digestive infections and 24.0% for the systemic infections. Infection with herpes simplex virus-1 and 2 is mostly seen during the pre-engraftment phase, whereas cytomegalovirus and HHV-6 are mainly found during the post-engraftment phase, and Epstein-Barr virus and varicella-zoster virus infections are often observed after the 100th day post-transplant (Sahin et al., 2016).

Yang et al. (2016) reported unexpected results indicating that mice treated with antiviral cocktail display more severe dextran sulfate sodium-induced colitis than untreated mice suggesting that gut resident viruses may insure maintenance of gut homeostasis. DCs isolated from colon of inflamed mice produced interferon-β in a TLR-dependent manner. When mice were reconstituted with toll-like receptors or rotavirus, colitis symptoms were significantly ameliorated, suggesting that enteric rotaviruses damped gut inflammation *via* toll-like receptors TLR-3 and TLR-7-mediated interferon β production. Consequently, it could not be excluded that human gut eukaryotic viruses can also be beneficial to patients in limiting GVHD. Legoff et al. (2017) reported that within a cohort of patients who received allo-HCT, patients who experienced enteric GVHD had both decreased richness of virome and higher abundance of *Microviridae*. *Anelloviridae*, *Polyomaviridae*, and *Herpesviridae* were found in immunocompromised allo-HCT patients likely related to viral reactivation. Recently, it was reported that myxoma virus, a *Poxviridae* that exhibits oncolytic activity against various hematologic malignancies like multiple myeloma or acute myeloid leukemia could be used, has a tool for *ex-vivo* treatment of allo-HCT with evidence of possible GVHD abrogation without impairing graft-versus-tumor effects against residual cancer cells (Villa and McFadden, 2018).

## Discussion

Allo-HCT is a potentially curative treatment of hematologic malignancies. However, the effectiveness of this treatment remains limited by the high incidence of acute GVHD, which is the principal cause of death in allo-HCT (Riwes and Reddy, 2018). Pioneers’ experimental studies carried out in the 1970s already indicated less acute GVHD in mice which received allo-HCT in germ-free conditions or experimental group receiving gut decontamination antibiotics (van Bekkum et al., 1974). The influence of intestinal bacteria on the development of acute GVHD was further confirmed in patients receiving allo-HCT as treatment of hematologic malignancies. More than 20 years ago, evidence was obtained that antimicrobial chemotherapy (metronidazole and ciprofloxacin) targeted to intestinal anaerobic bacteria in marrow transplant recipients significantly reduced the severity of acute GVHD (Beelen et al., 1999). Although it was hypothesized that allogenic GVHD is driven by initial interaction between APCs that encounter donor T lymphocytes that in turn release pro-inflammatory cytokines recruiting allo-reactive T cells, the molecular crosstalk that account for allo-HCT tolerance or GVHD was largely ignored (Magenau et al., 2016). Indeed, it is very likely that during the set up of post-transplant GVHD, the control of the innate immune response is dependent on both the bacteria which produce butyrate and those which produce lactase. The precise quantitative and qualitative adjustment of these bacterial populations is probably the key to the balance between immune tolerance and inflammatory process/GVHD.

A decrease in SCFAs (butyrate) was found to be associated with an increased mortality from GVHD following allo-HCT transplantation (Mathewson et al., 2016). Next, the same research group reported that the GVHD protective effect of SCFAs requires GPR43-mediated intracellular signaling that triggers ERK phosphorylation, ERK-dependent activation of IL-18, and activation of the NOD-LRR‐ and pyrin domain-containing protein 3 (NLRP3) inflammasome (Fujiwara et al., 2018). GPR43 is expressed by intestinal epithelial cells, as well as by antigen presenting cells (APCs, such as macrophages and DCs). Fujiwara et al. (2018), reported that macrophages and DCs isolated from ileum and colon of allo-HCT recipients show greater expression of GPR43 (whereas *in vivo* GPR43 expression on donor T cells is weak), but only the expression of GPR43 on non-hematopoietic cells (presumably intestinal epithelial cells) was involved in protective effect of SCFAs against GVHD. Moreover, a diet supplemented with butyrate mitigates GVHD. According to Golob et al. (2019), the beneficial response to butyrate would be biphasic with the first phase corresponding to the prevention of acute GVHD and the second phase dealing with the recovery, once colitis is durably established. It was also reported that the abundance of *Clostridia* decreased in the microbiota of allo-transplant patients that experienced GHVD and was accompanied by alteration in gastrointestinal microbiota-derived butyrate (Mathewson et al., 2016). The central role played by butyrate in the prevention of GVHD indicates that the presence of butyrogenic bacteria is essential for GVHD prevention. Butyrate is the main microbiota-derived regulator of gut mucosal immunity. Butyrogenic bacteria are considered oxidative stress sensitive. Most of these bacteria do not synthesize butyrate in the absence of cross-feeding bacteria that are oxidative stress sensitive. Under oxidative stress, the presence of butyrogenic bacteria in the microbiota decrease and oxidative stress-resistant strains (that encode enzymes such as catalase or superoxide dismutase required to deal with oxidative stress) are selected and trigger secretion of pro-inflammatory cytokines by immune response cells (Million et al., 2018).

In human, lactase persistence is associated with enhanced milk intake, higher waist circumference, and cardiometabolic abnormalities. Higher dairy intake was associated with higher body mass index supporting a causal link between lactose ingestion and body weight (Huang et al., 2018). When consuming dairy products, a person with lactase intolerance (the most common cause of the disease being a genetic mutation in the promoter of the gene that codes for lactase resulting in loss of intestinal lactase) may experience the symptoms of lactose intolerance which includes nausea, abdominal pain, gas, bloating, and diarrhea (Misselwitz et al., 2019; Kastl et al., 2020). Beside lactose, dairy products also provide numerous biologically active factors including growth factors, including the fibroblast growth factor FGF21 (mainly produced in the liver, brown and white adipocyte tissues, and pancreas) that was found to enhance MAPkinases (ERK1/2) phosphorylation in intestinal explants and to stimulate lactase production and lactose absorption (Gavaldà-Navarro et al., 2015). Gavaldà-Navarro et al. (2015) reported that mice feed with milk from FGF21 knockout mice showed decreased expression of lactase and maltase glucoamylase in the ileum. FGF21 binds to the FGFreceptor in complex with βKlotho to trigger cell signaling (Kurosu et al., 2007), yet the mode of FGF21 regulation of lactase expression by intestinal cells remains to be explored. This growth factor is known for stimulating the oxidation of fatty-acids, the production of ketone bodies, and inhibition of lipogenesis, thus regulating the glucose-lipid metabolism (Tezze et al., 2019). Recently, it was reported that feeding allo-transplanted mice with lactose free diet mitigated GVHD and reduced post-transplant *Enterococcus* proliferation (the optimal growth of which depends on lactose availability), whereas in mice feed with a dairy products diet, the lactase expression declined in the duodenum during the course of transplantation and induced a pathological state resembling lactose intolerance (Stein-Thoeringer et al., 2019).

Holler et al. (2014) reported a shift toward Enterococci after allo-HCT with a decrease in the obligate Firmicutes anaerobic bacteria that was much pronounced in the patients treated with antibiotics. Stein-Thoeringer et al. (2019) hypothesized that intestinal mucosal damages caused by irradiation or allo-reactive T cells may reduce the production of lactase from the small intestine enterocytes, that duodenal lactase progressively decline allowing undigested lactose to reach the lower intestinal tract where it serves as metabolic fuels for the growth of *Enterococcus* (e.g., *En. faecium* in human, *En. faecalis* in mice). Nalle et al. (2019) recently reported that MLCK210-deficient mice exhibited limited GVHD and were protected from epithelial barrier damages and CD8^+^ T cells proliferation. These authors discriminate the initiation of GVHD considered to be tight junction-independent from GVHD propagation that is MLCK210-dependent. Stein-Thoeringer et al. (2019) reported that *En. faecium* (only recently considered a human pathogen) dominate in patients with allo-HCT between 3-week and 3-month post-transplantation and that fecal domination by *Enterococcus* in the early post-transplant period (the first 3-week post-transplant) was associated with increased GVHD and overall mortality. They also found the VanA operon in a subset (37.4%) of patients. In an animal model, Stein-Thoeringer et al. (2019) reported a transient expansion of *En. faecalis* in GVHD mice and administration of *En. faecalis* aggravated the GVHD, thus demonstrating a direct link between the *Enterococcus* predominance and the GVHD. A major breakthrough came along with the attempt to demonstrate that the post-transplant defect of mucosal defense mechanisms facilitate the expansion of *Enterococcus*. They found that intestinal antimicrobial peptides of the Reg3 family, known to suppress the growth of vancomycin resistant Enterococci (VRE bacteria) that exploit innate immune deficits (Brandl et al., 2008), were reduced in the ileum of GVHD mice. Obviously, the primary strategy to prevent GVHD after allo-HCT is immunosuppression, but such treatment may increase the risk of enteropathogenic bacteria invasion. Stein-Thoeringer et al. (2019) also provided an elegant demonstration that *En. faecium*-dominated microbiota observed after HCT were enriched in bioactive compounds involved in lactose and galactose degradation pathway (with similar result in *En. faecalis*-dominated microbiota in mice). *En. faecalis* expresses the gelatinase (GelE) a matrix metalloprotease, so-called sheddase, that cleaves E-cadherin (Steck et al., 2011) and activates the protease-activated receptor 2 (PARP2), a transmembrane G-protein-coupled receptor (Maharshak et al., 2015). These molecules contribute to the disruption of intestinal barriers and inflammation. According to the UniProt database^1^, *En. faecalis* also encodes a predicted htrA protein serine protease, another candidate sheddase (Devaux et al., 2019).

In mice animal model, it was observed that PPIs (namely omeprazole) administered to mice cause reduced gastric secretion and favor the expansion of *Enterococcus* species including *En. faecalis* (Llorente et al., 2017). This favor bacterial translocation in mesenteric lymph nodes and the liver with subsequent inflammation, hepatocytes death, and non-alcoholic steatohepatosis. Mice that lack expression of the TLR2 (a cell membrane receptor that recognizes peptidoglycan from Gram-positive bacteria) or the myeloid differentiation primary response 88, MYD88 (intracellular adaptor molecule for TLRs), lack innate immune response and were protected from *En. faecalis*-induced inflammation, steatosis, and liver injury. *En. faecalis* was found capable of suppressing the innate immune response of macrophages through repression of NF-κB signaling (Tien et al., 2017). In a human cohort, administration of omeprazole for preclinical evaluation, the frequency of *Enterococcus* in the fecal samples was increased after 2-week of PPI treatment. This corroborates previous observation indicating that an omeprazole therapy in cirrhosis demonstrated a shift in fecal microbiota composition (Bajaj et al., 2014; Merli et al., 2015). Innate immune response that follows the engagement of TLRs is involved in the process of GVHD. The TLR5 agonist fagellin was shown to reduce GVHD and preserve long post-transplant immune reconstitution characterized by more Fop-3^+^ T regulator (T reg) cells (Hossain et al., 2011). Although it remains to be documented in humans suffering from ten-eleven translocation (TET) methylcytosine dioxygenase 2 (*TET2*)-deficiency, in an animal model it was reported that mutations in *TET2*, which encodes an epigenetic modifier enzyme that influence the activation of regulatory CD4^+^ T cells (T reg cells) *via* FoxP3 (Yue et al., 2016), can be associated with dysfunction of the small intestine barrier, bacterial translocation and IL-6 production known to be a critical activator of myelopoiesis in response to systemic bacterial dissemination (Meisel et al., 2018).

*Clostridium difficile* has also been implicated as a potential trigger of immune reaction that can contribute to the development of GVHD. Working with a cohort of 75 allo-HCT patients, Chakarbati et al. (2000), found that *C. difficile* was frequently associated with severe GVHD (grade 3–4) and the presence of the bacteria in stool worsened the pathophysiology of GVHD in 60% of patients. The relationship between the presence of *C. difficile* and GVHD was also reported by Dubberke et al. (2007) who reported that patients with *C. difficile* showed a higher propensity to develop new-onset GVHD and severe forms of GVHD. Similar observations were reported from the investigation of a cohort of 822 allo-HCT patients regarding the risk to develop severe GVHD at day 60 and day 100 after allo-HCT when *C. difficile* was present in the patients (Trifilio et al., 2012). The incidence was more than 20% in patients who were older than 60 years and carried a vancomycin-resistant (VRE) *C. difficile*. Indeed, *C. difficile* positive diagnosis was found to precede GVHD diagnosis in 85.7% of allo-HCT patients who developed gut GVHD while the overall 1-year incidence of *C. difficile* was 9.2% among a cohort of 999 allo-HCT patients (Alonso et al., 2012; Alonso and Marr, 2013). Although the presence of VRE *C. difficile* was not investigated in the main papers discussed in this review, it can be hypothesized that the decreased microbiota diversity (butyrogenic bacteria) facilitates the clonal expansion *C. difficile* strains in the gut of these patients (Hocquart et al., 2018), which in turn represents an aggravating factor regarding the risk of mortality by GVHD of allo-HCT patients.

CTLA-4, currently known as a target for immune checkpoint inhibitor antibodies, was originally described as a T cell surface molecule that negatively influence immune response by competing with CD28 for binding to ligands on APCs. In patients with severe alcoholic hepatitis, it was reported that PD-1 immune checkpoint-receptor inhibition restored the adaptive antibacterial T cell response which otherwise was defective (Markwick et al., 2015). The approval of immune checkpoint inhibitors (such as CTLA-4 or PD1/PD-L1 inhibitor antibodies) in the treatment of cancers has changed the outcome of several of such severe diseases and highlighted the strong molecular crosstalk between the microbiota and the immune system (Zitvogel et al., 2018; Gong et al., 2019). Defects in the microbiota can compromise the therapeutic efficacy or have secondary side effect on the gut. In melanoma therapy, anti-PD1 and anti-CTLA-4 antibodies have been approved. However, immune-mediated colitis in patients with melanoma after dual checkpoint inhibitors treatment was described (Thalambedu et al., 2019). A variety of genera can reduce the immune checkpoint inhibitor-induced colitis possibly by limiting the inflammation through expansion of CD4^+^/FoxP3^+^ T reg cells and/or production of anti-inflammatory cytokines (Dubin et al., 2016). In a murine model of melanoma, CD8^+^ T cell activation in response to PD-L1 inhibitor correlated with mice, which received fecal transplantations from patients abundant in bacteria from the Ruminococcaceae family and *Faecalibacterium* spp., whereas nonresponders were characterized by elevated presence of CD4^+^/FoxP3^+^ T reg cells and received stools which were abundant in Bacteroidales (Gopalakrishnan et al., 2018).

The experience accumulated in the field of cancer therapy with immune checkpoint inhibitors provides evidence that the microbiota govern the balance between CD8^+^ T cell activation and inflammation on one side and CD4^+^/FoxP3^+^ T reg cells and lack of inflammation on the other side. It can be extrapolated that high butyrate allows CD4^+^/FoxP3^+^ T reg cells expansion and prevents inflammation, and GHVD reaction whereas low lactase induces lactic acid bacteria expansion, CD8^+^ T cell activation, inflammation and GVHD. However, we are just beginning to understand what could be the role of the microbiota and the impact of cross feeding in GVHD. First, variation in the composition of the microbiota between allo-HCT patients that lack GVHD and those who unfortunately experience GVHD is currently well established. Second, the role played by butyrogenic and lactic acid bacteria begun to be understood. However, it is now necessary to enter more deeply into the characterization of the bacterial species that distinguish homeostasis from dysbiosis in non-GVHD versus GVHD patients and to study their enzymatic equipment which can vary both at the level of the bacterial strain and at the clone level. There is currently evidence that the adverse outcomes of irradiation and antibiotics/chemotherapy treatments (oxidative stress) on gut microbiota could be avoided by using probiotics and/or fecal microbiota transplant, opening new perspective for the prevention of GVHD in allo-HCT patients (Delia et al., 2002; Kaito et al., 2018; Severyn et al., 2019; Shouval et al., 2019). The next challenge is to offer transplant patients a beneficial combination of easy-to-consume probiotics, which should prevent the occurrence of GVHD. Strict anaerobic bacteria are not the easiest to cultivate and produce in large quantity for therapeutic application. We have recently reported that oxygen-sensitive bacteria can maintain butyrate production despite the presence of oxygen when treated with antioxidants (Million et al., 2020). Among lactic acid bacteria, the *Bifidobacterium* seem to be the best candidates as anti-GVHD probiotics. In contrast, the Enterococci which also metabolize lactose are suspected of being deleterious and responsible for the production of noxious substances. If this therapeutic cocktail must contain cross feeding bacteria and butyrogenic bacteria, it will be necessary to exclude those which are known for their deleterious side effects (i.e., *Clostridium butyricum*, associated with enterocolitis, or *Mediterraneibacter gnavus*, associated with obesity) and to investigate the beneficial cooperative effects of bacteria such as *B. adolescentis*, *B. longum*, *Eu. hallii*, *R. intestinalis*, and particularly *F. prausnitzii*.

## Author Contributions

CD, MM, and DR contributed to the conception of the manuscript. CD wrote the manuscript. All authors contributed to the article and approved the submitted version.

### Conflict of Interest

The authors declare that the research was conducted in the absence of any commercial or financial relationships that could be construed as a potential conflict of interest.

## Footnotes

^1^https://www.uniprot.org/uniprot/Q82ZM6
